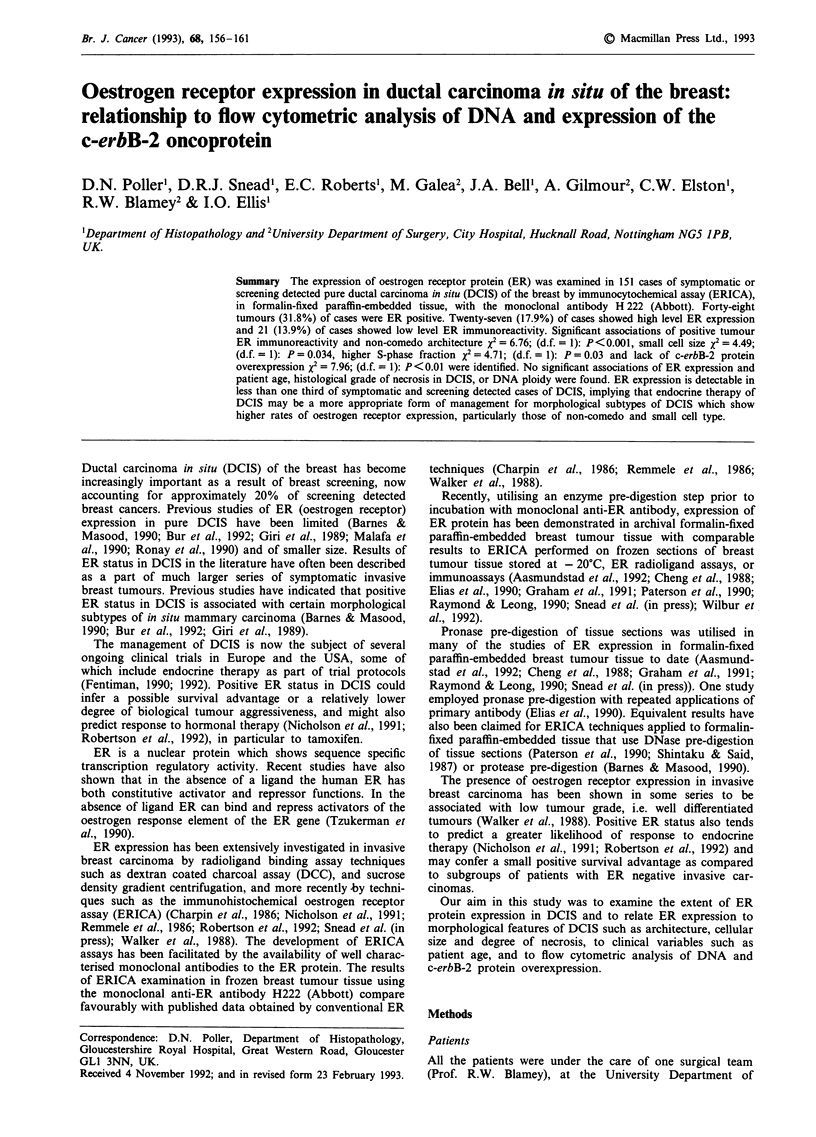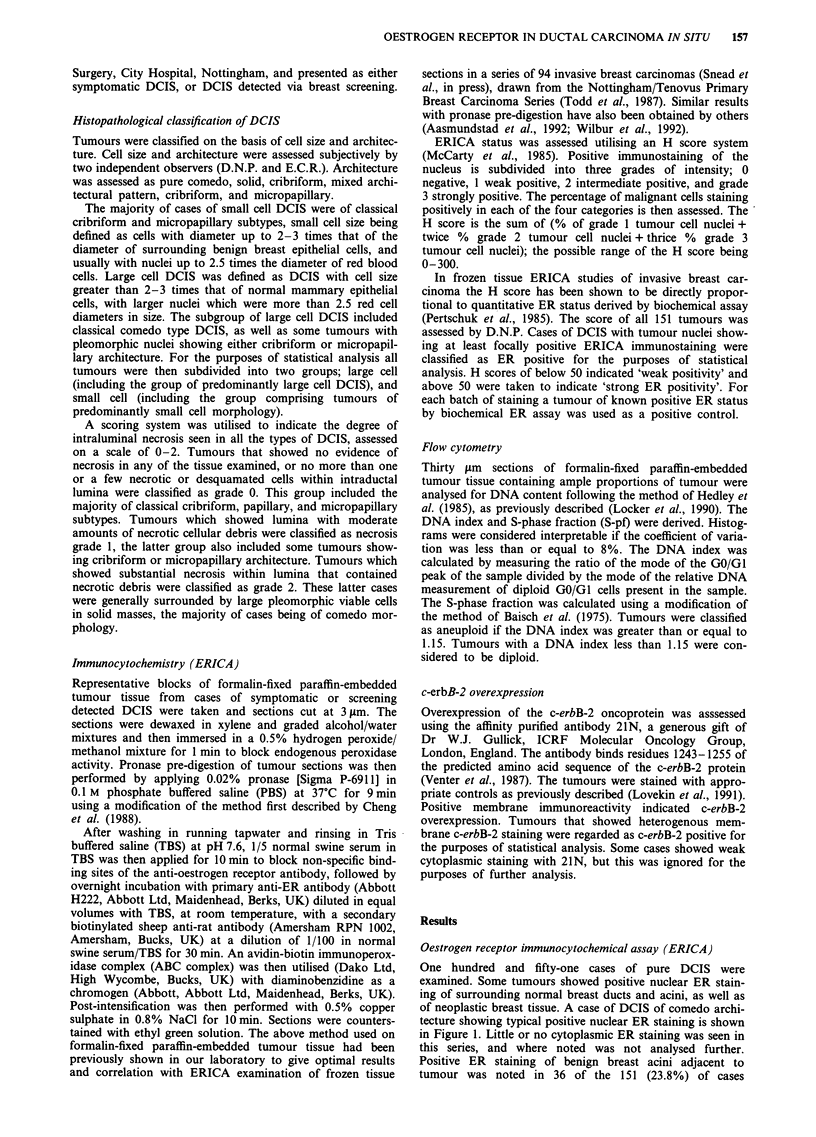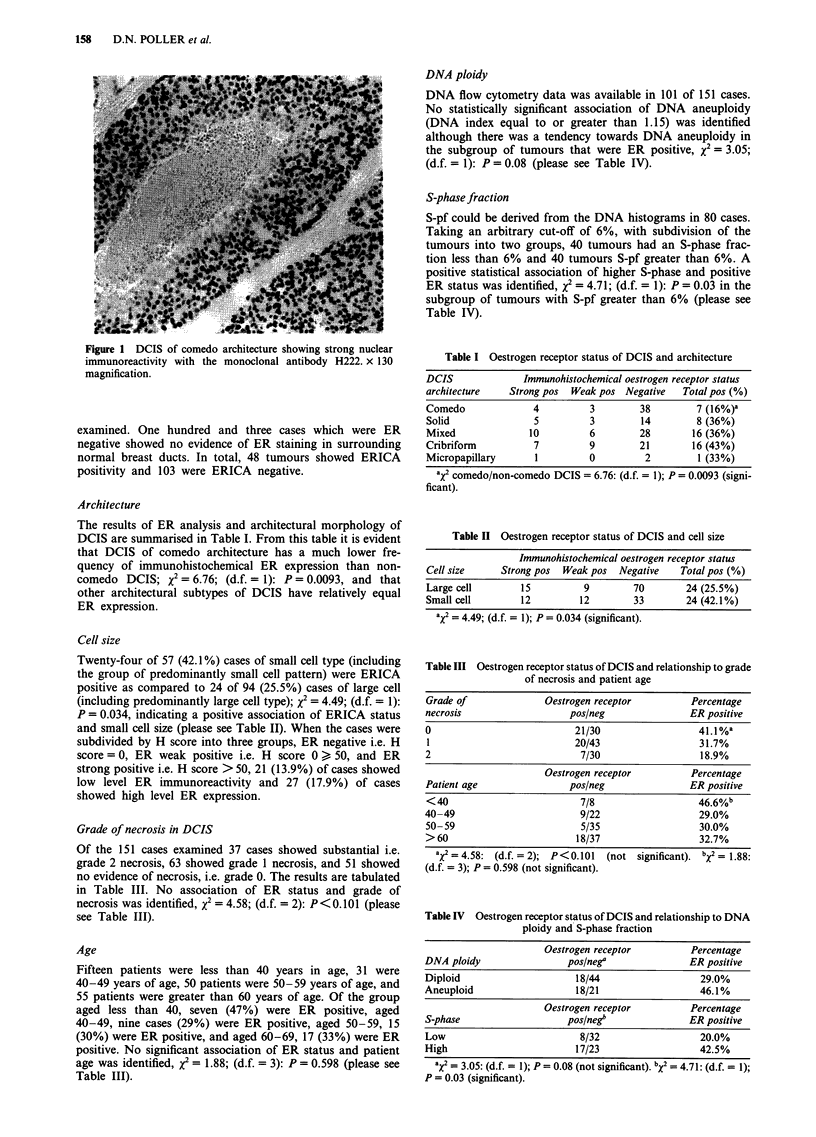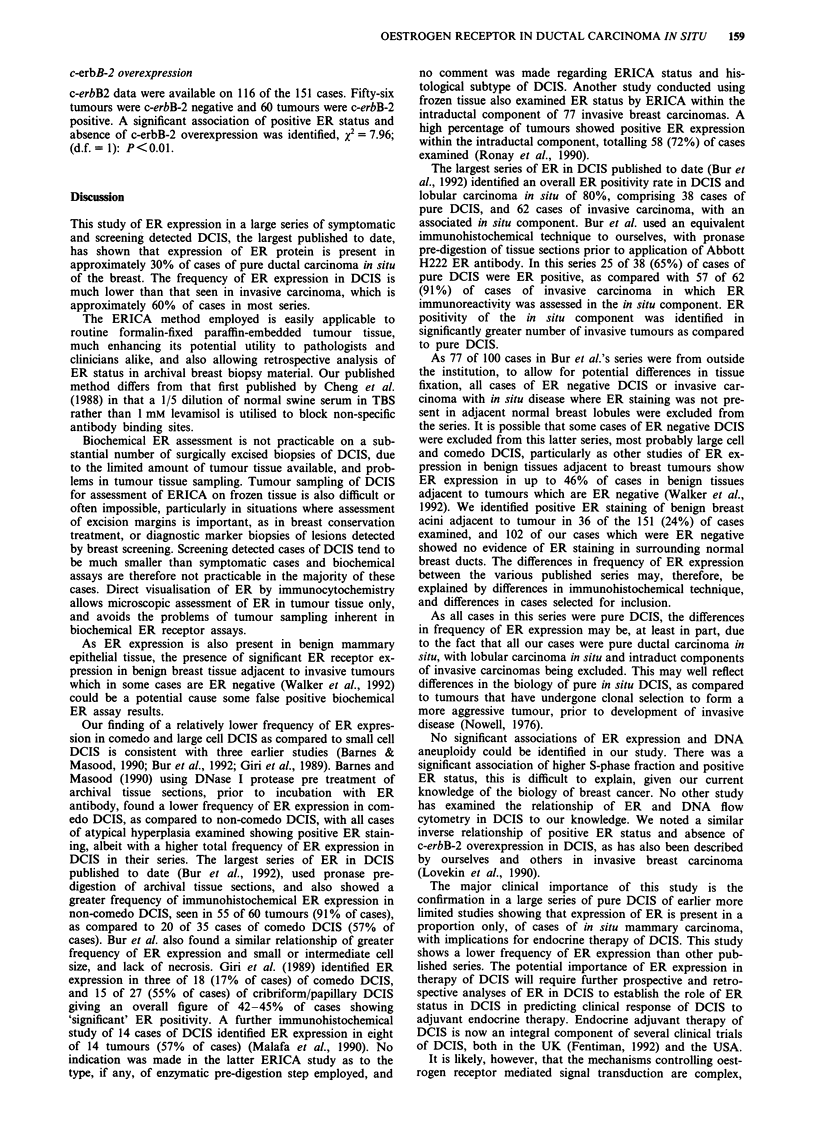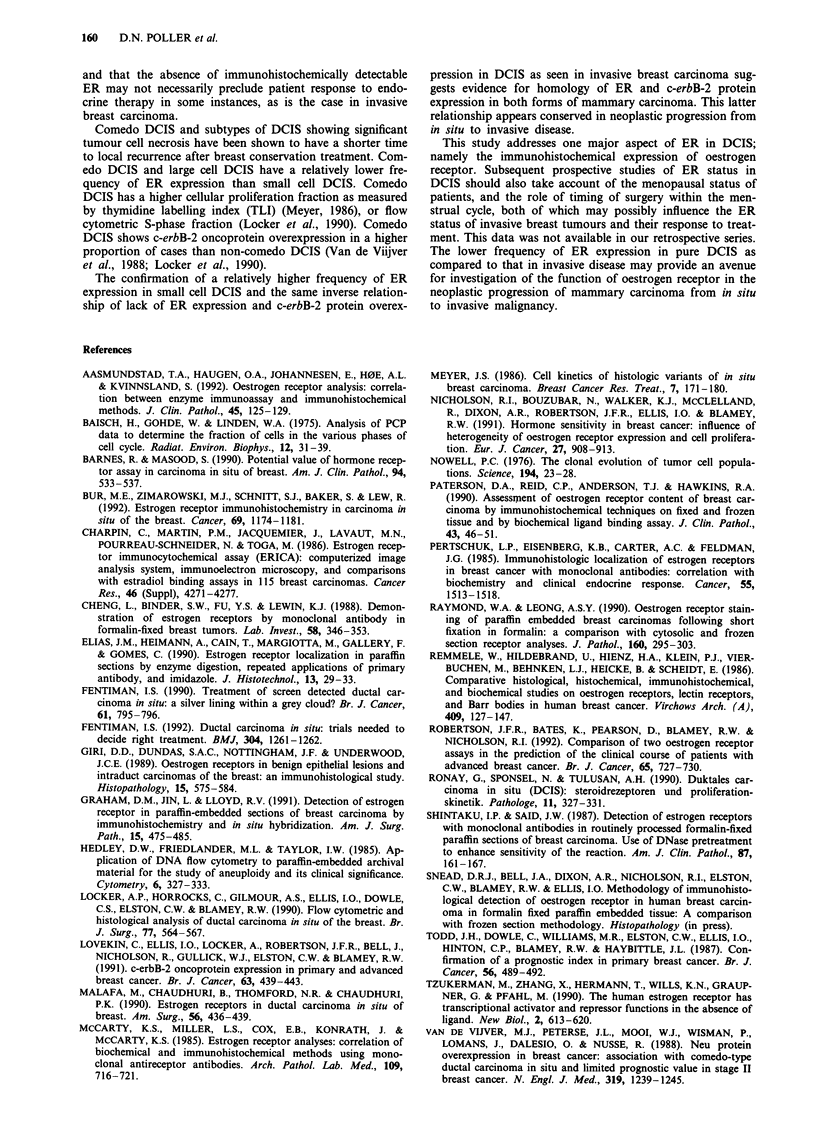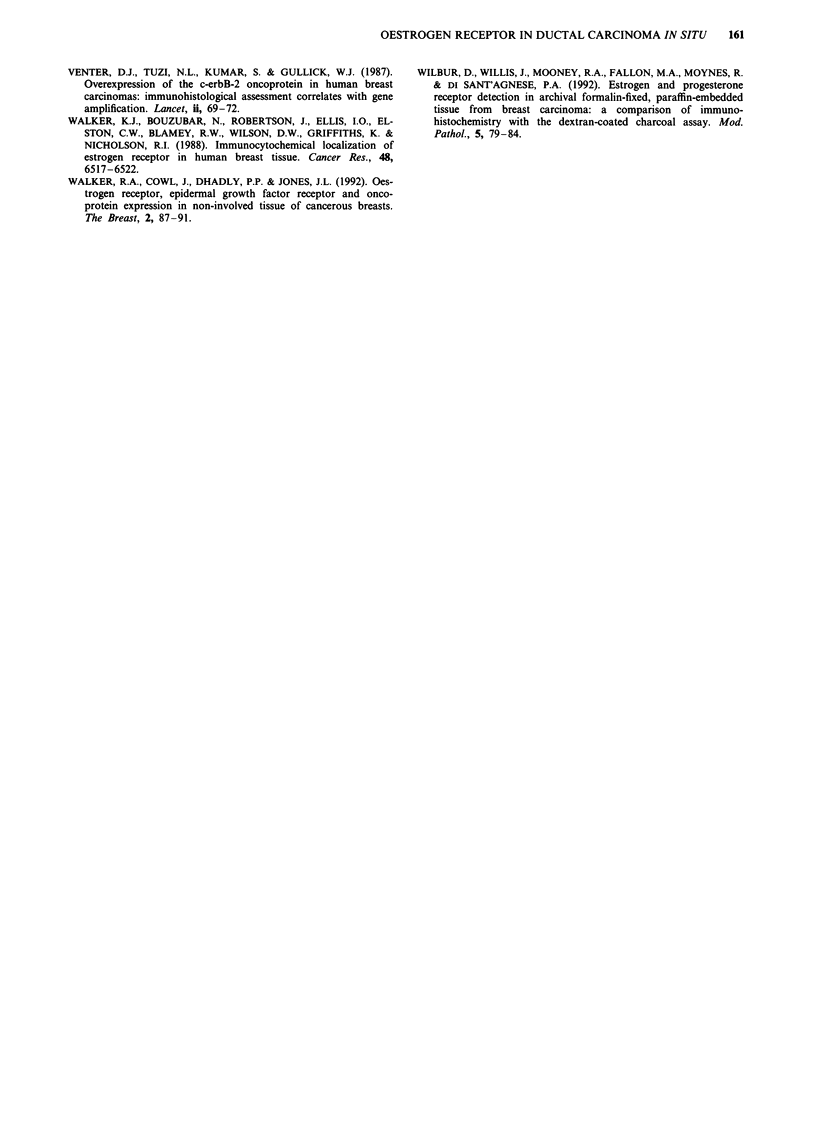# Oestrogen receptor expression in ductal carcinoma in situ of the breast: relationship to flow cytometric analysis of DNA and expression of the c-erbB-2 oncoprotein.

**DOI:** 10.1038/bjc.1993.305

**Published:** 1993-07

**Authors:** D. N. Poller, D. R. Snead, E. C. Roberts, M. Galea, J. A. Bell, A. Gilmour, C. W. Elston, R. W. Blamey, I. O. Ellis

**Affiliations:** Department of Histopathology, City Hospital, Nottingham, UK.

## Abstract

**Images:**


Br. J. Cancer (1993), 68, 156-161 © Macmillan Press Ltd., 1993

### Oestrogen receptor expression in ductal carcinoma *in situ* of the breast: relationship to flow cytometric analysis of DNA and expression of the c-*erb*B-2 oncoprotein

D.N. Poller[1], D.R.J. Snead[1], E.C. Roberts[1], M. Galea[2], J.A. Bell[1], A. Gilmour[2], C.W. Elston[1], R.W. Blamey[2] & I.O. Ellis[1]

[1]*Department of Histopathology and* [2]*University Department of Surgery, City Hospital, Hucknall Road, Nottingham NG5 1PB, UK.*

**Summary** The expression of oestrogen receptor protein (ER) was examined in 151 cases of symptomatic or screening detected pure ductal carcinoma *in situ* (DCIS) of the breast by immunocytochemical assay (ERICA), in formalin-fixed paraffin-embedded tissue, with the monoclonal antibody H 222 (Abbott). Forty-eight tumours (31.8%) of cases were ER positive. Twenty-seven (17.9%) of cases showed high level ER expression and 21 (13.9%) of cases showed low level ER immunoreactivity. Significant associations of positive tumour ER immunoreactivity and non-comedo architecture $\chi^2 = 6.76$; (d.f. = 1): $P < 0.001$, small cell size $\chi^2 = 4.49$; (d.f. = 1): $P = 0.034$, higher S-phase fraction $\chi^2 = 4.71$; (d.f. = 1): $P = 0.03$ and lack of c-*erb*B-2 protein overexpression $\chi^2 = 7.96$; (d.f. = 1): $P < 0.01$ were identified. No significant associations of ER expression and patient age, histological grade of necrosis in DCIS, or DNA ploidy were found. ER expression is detectable in less than one third of symptomatic and screening detected cases of DCIS, implying that endocrine therapy of DCIS may be a more appropriate form of management for morphological subtypes of DCIS which show higher rates of oestrogen receptor expression, particularly those of non-comedo and small cell type.

Ductal carcinoma *in situ* (DCIS) of the breast has become increasingly important as a result of breast screening, now accounting for approximately 20% of screening detected breast cancers. Previous studies of ER (oestrogen receptor) expression in pure DCIS have been limited (Barnes & Masood, 1990; Bur *et al.*, 1992; Giri *et al.*, 1989; Malafa *et al.*, 1990; Ronay *et al.*, 1990) and of smaller size. Results of ER status in DCIS in the literature have often been described as a part of much larger series of symptomatic invasive breast tumours. Previous studies have indicated that positive ER status in DCIS is associated with certain morphological subtypes of *in situ* mammary carcinoma (Barnes & Masood, 1990; Bur *et al.*, 1992; Giri *et al.*, 1989).

The management of DCIS is now the subject of several ongoing clinical trials in Europe and the USA, some of which include endocrine therapy as part of trial protocols (Fentiman, 1990; 1992). Positive ER status in DCIS could infer a possible survival advantage or a relatively lower degree of biological tumour aggressiveness, and might also predict response to hormonal therapy (Nicholson *et al.*, 1991; Robertson *et al.*, 1992), in particular to tamoxifen.

ER is a nuclear protein which shows sequence specific transcription regulatory activity. Recent studies have also shown that in the absence of a ligand the human ER has both constitutive activator and repressor functions. In the absence of ligand ER can bind and repress activators of the oestrogen response element of the ER gene (Tzukerman *et al.*, 1990).

ER expression has been extensively investigated in invasive breast carcinoma by radioligand binding assay techniques such as dextran coated charcoal assay (DCC), and sucrose density gradient centrifugation, and more recently by techniques such as the immunohistochemical oestrogen receptor assay (ERICA) (Charpin *et al.*, 1986; Nicholson *et al.*, 1991; Remmele *et al.*, 1986; Robertson *et al.*, 1992; Snead *et al.* (in press); Walker *et al.*, 1988). The development of ERICA assays has been facilitated by the availability of well characterised monoclonal antibodies to the ER protein. The results of ERICA examination in frozen breast tumour tissue using the monoclonal anti-ER antibody H222 (Abbott) compare favourably with published data obtained by conventional ER techniques (Charpin *et al.*, 1986; Remmele *et al.*, 1986; Walker *et al.*, 1988).

Correspondence: D.N. Poller, Department of Histopathology, Gloucestershire Royal Hospital, Great Western Road, Gloucester GL1 3NN, UK.
Received 4 November 1992; and in revised form 23 February 1993.

Recently, utilising an enzyme pre-digestion step prior to incubation with monoclonal anti-ER antibody, expression of ER protein has been demonstrated in archival formalin-fixed paraffin-embedded breast tumour tissue with comparable results to ERICA performed on frozen sections of breast tumour tissue stored at − 20°C, ER radioligand assays, or immunoassays (Aasmundstad *et al.*, 1992; Cheng *et al.*, 1988; Elias *et al.*, 1990; Graham *et al.*, 1991; Paterson *et al.*, 1990; Raymond & Leong, 1990; Snead *et al.* (in press); Wilbur *et al.*, 1992).

Pronase pre-digestion of tissue sections was utilised in many of the studies of ER expression in formalin-fixed paraffin-embedded breast tumour tissue to date (Aasmundstad *et al.*, 1992; Cheng *et al.*, 1988; Graham *et al.*, 1991; Raymond & Leong, 1990; Snead *et al.* (in press)). One study employed pronase pre-digestion with repeated applications of primary antibody (Elias *et al.*, 1990). Equivalent results have also been claimed for ERICA techniques applied to formalin-fixed paraffin-embedded tissue that use DNase pre-digestion of tissue sections (Paterson *et al.*, 1990; Shintaku & Said, 1987) or protease pre-digestion (Barnes & Masood, 1990).

The presence of oestrogen receptor expression in invasive breast carcinoma has been shown in some series to be associated with low tumour grade, i.e. well differentiated tumours (Walker *et al.*, 1988). Positive ER status also tends to predict a greater likelihood of response to endocrine therapy (Nicholson *et al.*, 1991; Robertson *et al.*, 1992) and may confer a small positive survival advantage as compared to subgroups of patients with ER negative invasive carcinomas.

Our aim in this study was to examine the extent of ER protein expression in DCIS and to relate ER expression to morphological features of DCIS such as architecture, cellular size and degree of necrosis, to clinical variables such as patient age, and to flow cytometric analysis of DNA and c-*erb*B-2 protein overexpression.

### Methods

### *Patients*

All the patients were under the care of one surgical team (Prof. R.W. Blamey), at the University Department of

Surgery, City Hospital, Nottingham, and presented as either symptomatic DCIS, or DCIS detected via breast screening.

*Histopathological classification of DCIS*

Tumours were classified on the basis of cell size and architecture. Cell size and architecture were assessed subjectively by two independent observers (D.N.P. and E.C.R.). Architecture was assessed as pure comedo, solid, cribriform, mixed architectural pattern, cribriform, and micropapillary.

The majority of cases of small cell DCIS were of classical cribriform and micropapillary subtypes, small cell size being defined as cells with diameter up to 2–3 times that of the diameter of surrounding benign breast epithelial cells, and usually with nuclei up to 2.5 times the diameter of red blood cells. Large cell DCIS was defined as DCIS with cell size greater than 2–3 times that of normal mammary epithelial cells, with larger nuclei which were more than 2.5 red cell diameters in size. The subgroup of large cell DCIS included classical comedo type DCIS, as well as some tumours with pleomorphic nuclei showing either cribriform or micropapillary architecture. For the purposes of statistical analysis all tumours were then subdivided into two groups; large cell (including the group of predominantly large cell DCIS), and small cell (including the group comprising tumours of predominantly small cell morphology).

A scoring system was utilised to indicate the degree of intraluminal necrosis seen in all the types of DCIS, assessed on a scale of 0–2. Tumours that showed no evidence of necrosis in any of the tissue examined, or no more than one or a few necrotic or desquamated cells within intraductal lumina were classified as grade 0. This group included the majority of classical cribriform, papillary, and micropapillary subtypes. Tumours which showed lumina with moderate amounts of necrotic cellular debris were classified as necrosis grade 1, the latter group also included some tumours showing cribriform or micropapillary architecture. Tumours which showed substantial necrosis within lumina that contained necrotic debris were classified as grade 2. These latter cases were generally surrounded by large pleomorphic viable cells in solid masses, the majority of cases being of comedo morphology.

*Immunocytochemistry (ERICA)*

Representative blocks of formalin-fixed paraffin-embedded tumour tissue from cases of symptomatic or screening detected DCIS were taken and sections cut at 3 μm. The sections were dewaxed in xylene and graded alcohol/water mixtures and then immersed in a 0.5% hydrogen peroxide/methanol mixture for 1 min to block endogenous peroxidase activity. Pronase pre-digestion of tumour sections was then performed by applying 0.02% pronase [Sigma P-6911] in 0.1 M phosphate buffered saline (PBS) at 37°C for 9 min using a modification of the method first described by Cheng *et al.* (1988).

After washing in running tapwater and rinsing in Tris buffered saline (TBS) at pH 7.6, 1/5 normal swine serum in TBS was then applied for 10 min to block non-specific binding sites of the anti-oestrogen receptor antibody, followed by overnight incubation with primary anti-ER antibody (Abbott H222, Abbott Ltd, Maidenhead, Berks, UK) diluted in equal volumes with TBS, at room temperature, with a secondary biotinylated sheep anti-rat antibody (Amersham RPN 1002, Amersham, Bucks, UK) at a dilution of 1/100 in normal swine serum/TBS for 30 min. An avidin-biotin immunoperoxidase complex (ABC complex) was then utilised (Dako Ltd, High Wycombe, Bucks, UK) with diaminobenzidine as a chromogen (Abbott, Abbott Ltd, Maidenhead, Berks, UK). Post-intensification was then performed with 0.5% copper sulphate in 0.8% NaCl for 10 min. Sections were counterstained with ethyl green solution. The above method used on formalin-fixed paraffin-embedded tumour tissue had been previously shown in our laboratory to give optimal results and correlation with ERICA examination of frozen tissue sections in a series of 94 invasive breast carcinomas (Snead *et al.*, in press), drawn from the Nottingham/Tenovus Primary Breast Carcinoma Series (Todd *et al.*, 1987). Similar results with pronase pre-digestion have also been obtained by others (Aasmundstad *et al.*, 1992; Wilbur *et al.*, 1992).

ERICA status was assessed utilising an H score system (McCarty *et al.*, 1985). Positive immunostaining of the nucleus is subdivided into three grades of intensity; 0 negative, 1 weak positive, 2 intermediate positive, and grade 3 strongly positive. The percentage of malignant cells staining positively in each of the four categories is then assessed. The H score is the sum of (% of grade 1 tumour cell nuclei + twice % grade 2 tumour cell nuclei + thrice % grade 3 tumour cell nuclei); the possible range of the H score being 0–300.

In frozen tissue ERICA studies of invasive breast carcinoma the H score has been shown to be directly proportional to quantitative ER status derived by biochemical assay (Pertschuk *et al.*, 1985). The score of all 151 tumours was assessed by D.N.P. Cases of DCIS with tumour nuclei showing at least focally positive ERICA immunostaining were classified as ER positive for the purposes of statistical analysis. H scores of below 50 indicated 'weak positivity' and above 50 were taken to indicate 'strong ER positivity'. For each batch of staining a tumour of known positive ER status by biochemical ER assay was used as a positive control.

*Flow cytometry*

Thirty μm sections of formalin-fixed paraffin-embedded tumour tissue containing ample proportions of tumour were analysed for DNA content following the method of Hedley *et al.* (1985), as previously described (Locker *et al.*, 1990). The DNA index and S-phase fraction (S-pf) were derived. Histograms were considered interpretable if the coefficient of variation was less than or equal to 8%. The DNA index was calculated by measuring the ratio of the mode of the G0/G1 peak of the sample divided by the mode of the relative DNA measurement of diploid G0/G1 cells present in the sample. The S-phase fraction was calculated using a modification of the method of Baisch *et al.* (1975). Tumours were classified as aneuploid if the DNA index was greater than or equal to 1.15. Tumours with a DNA index less than 1.15 were considered to be diploid.

*c-*erb*B-2 overexpression*

Overexpression of the c-*erb*B-2 oncoprotein was asssessed using the affinity purified antibody 21N, a generous gift of Dr W.J. Gullick, ICRF Molecular Oncology Group, London, England. The antibody binds residues 1243–1255 of the predicted amino acid sequence of the c-*erb*B-2 protein (Venter *et al.*, 1987). The tumours were stained with appropriate controls as previously described (Lovekin *et al.*, 1991). Positive membrane immunoreactivity indicated c-*erb*B-2 overexpression. Tumours that showed heterogenous membrane c-*erb*B-2 staining were regarded as c-*erb*B-2 positive for the purposes of statistical analysis. Some cases showed weak cytoplasmic staining with 21N, but this was ignored for the purposes of further analysis.

### Results

*Oestrogen receptor immunocytochemical assay (ERICA)*

One hundred and fifty-one cases of pure DCIS were examined. Some tumours showed positive nuclear ER staining of surrounding normal breast ducts and acini, as well as of neoplastic breast tissue. A case of DCIS of comedo architecture showing typical positive nuclear ER staining is shown in Figure 1. Little or no cytoplasmic ER staining was seen in this series, and where noted was not analysed further. Positive ER staining of benign breast acini adjacent to tumour was noted in 36 of the 151 (23.8%) of cases

**Figure 1** DCIS of comedo architecture showing strong nuclear immunoreactivity with the monoclonal antibody H222. × 130 magnification.

examined. One hundred and three cases which were ER negative showed no evidence of ER staining in surrounding normal breast ducts. In total, 48 tumours showed ERICA positivity and 103 were ERICA negative.

*Architecture*

The results of ER analysis and architectural morphology of DCIS are summarised in Table I. From this table it is evident that DCIS of comedo architecture has a much lower frequency of immunohistochemical ER expression than non-comedo DCIS; $\chi^2 = 6.76$; (d.f. = 1): $P = 0.0093$, and that other architectural subtypes of DCIS have relatively equal ER expression.

*Cell size*

Twenty-four of 57 (42.1%) cases of small cell type (including the group of predominantly small cell pattern) were ERICA positive as compared to 24 of 94 (25.5%) cases of large cell (including predominantly large cell type); $\chi^2 = 4.49$; (d.f. = 1): $P = 0.034$, indicating a positive association of ERICA status and small cell size (please see Table II). When the cases were subdivided by H score into three groups, ER negative i.e. H score = 0, ER weak positive i.e. H score $0 \geqslant 50$, and ER strong positive i.e. H score $>50$, 21 (13.9%) of cases showed low level ER immunoreactivity and 27 (17.9%) of cases showed high level ER expression.

*Grade of necrosis in DCIS*

Of the 151 cases examined 37 cases showed substantial i.e. grade 2 necrosis, 63 showed grade 1 necrosis, and 51 showed no evidence of necrosis, i.e. grade 0. The results are tabulated in Table III. No association of ER status and grade of necrosis was identified, $\chi^2 = 4.58$; (d.f. = 2): $P < 0.101$ (please see Table III).

*Age*

Fifteen patients were less than 40 years in age, 31 were 40–49 years of age, 50 patients were 50–59 years of age, and 55 patients were greater than 60 years of age. Of the group aged less than 40, seven (47%) were ER positive, aged 40–49, nine cases (29%) were ER positive, aged 50–59, 15 (30%) were ER positive, and aged 60–69, 17 (33%) were ER positive. No significant association of ER status and patient age was identified, $\chi^2 = 1.88$; (d.f. = 3): $P = 0.598$ (please see Table III).

*DNA ploidy*

DNA flow cytometry data was available in 101 of 151 cases. No statistically significant association of DNA aneuploidy (DNA index equal to or greater than 1.15) was identified although there was a tendency towards DNA aneuploidy in the subgroup of tumours that were ER positive, $\chi^2 = 3.05$; (d.f. = 1): $P = 0.08$ (please see Table IV).

*S-phase fraction*

S-pf could be derived from the DNA histograms in 80 cases. Taking an arbitrary cut-off of 6%, with subdivision of the tumours into two groups, 40 tumours had an S-phase fraction less than 6% and 40 tumours S-pf greater than 6%. A positive statistical association of higher S-phase and positive ER status was identified, $\chi^2 = 4.71$; (d.f. = 1): $P = 0.03$ in the subgroup of tumours with S-pf greater than 6% (please see Table IV).

**Table I** Oestrogen receptor status of DCIS and architecture

| *DCIS architecture* | *Immunohistochemical oestrogen receptor status* Strong pos | Weak pos | Negative | Total pos (%) |
|---|---|---|---|---|
| Comedo | 4 | 3 | 38 | 7 (16%)[a] |
| Solid | 5 | 3 | 14 | 8 (36%) |
| Mixed | 10 | 6 | 28 | 16 (36%) |
| Cribriform | 7 | 9 | 21 | 16 (43%) |
| Micropapillary | 1 | 0 | 2 | 1 (33%) |

[a]$\chi^2$ comedo/non-comedo DCIS = 6.76: (d.f. = 1); $P = 0.0093$ (significant).

**Table II** Oestrogen receptor status of DCIS and cell size

| *Cell size* | *Immunohistochemical oestrogen receptor status* Strong pos | Weak pos | Negative | Total pos (%) |
|---|---|---|---|---|
| Large cell | 15 | 9 | 70 | 24 (25.5%) |
| Small cell | 12 | 12 | 33 | 24 (42.1%) |

[a]$\chi^2 = 4.49$; (d.f. = 1); $P = 0.034$ (significant).

**Table III** Oestrogen receptor status of DCIS and relationship to grade of necrosis and patient age

| *Grade of necrosis* | *Oestrogen receptor pos/neg* | *Percentage ER positive* |
|---|---|---|
| 0 | 21/30 | 41.1%[a] |
| 1 | 20/43 | 31.7% |
| 2 | 7/30 | 18.9% |
| *Patient age* | *Oestrogen receptor pos/neg* | *Percentage ER positive* |
| <40 | 7/8 | 46.6%[b] |
| 40–49 | 9/22 | 29.0% |
| 50–59 | 5/35 | 30.0% |
| >60 | 18/37 | 32.7% |

[a]$\chi^2 = 4.58$: (d.f. = 2); $P < 0.101$ (not significant). [b]$\chi^2 = 1.88$: (d.f. = 3); $P = 0.598$ (not significant).

**Table IV** Oestrogen receptor status of DCIS and relationship to DNA ploidy and S-phase fraction

| *DNA ploidy* | *Oestrogen receptor pos/neg[a]* | *Percentage ER positive* |
|---|---|---|
| Diploid | 18/44 | 29.0% |
| Aneuploid | 18/21 | 46.1% |
| *S-phase* | *Oestrogen receptor pos/neg[b]* | *Percentage ER positive* |
| Low | 8/32 | 20.0% |
| High | 17/23 | 42.5% |

[a]$\chi^2 = 3.05$: (d.f. = 1); $P = 0.08$ (not significant). [b]$\chi^2 = 4.71$: (d.f. = 1); $P = 0.03$ (significant).

*c-*erb*B-2 overexpression*

c-*erb*B2 data were available on 116 of the 151 cases. Fifty-six tumours were c-*erb*B-2 negative and 60 tumours were c-*erb*B-2 positive. A significant association of positive ER status and absence of c-erbB-2 overexpression was identified, $\chi^2 = 7.96$; (d.f. = 1): $P < 0.01$.

### Discussion

This study of ER expression in a large series of symptomatic and screening detected DCIS, the largest published to date, has shown that expression of ER protein is present in approximately 30% of cases of pure ductal carcinoma *in situ* of the breast. The frequency of ER expression in DCIS is much lower than that seen in invasive carcinoma, which is approximately 60% of cases in most series.

The ERICA method employed is easily applicable to routine formalin-fixed paraffin-embedded tumour tissue, much enhancing its potential utility to pathologists and clinicians alike, and also allowing retrospective analysis of ER status in archival breast biopsy material. Our published method differs from that first published by Cheng *et al.* (1988) in that a 1/5 dilution of normal swine serum in TBS rather than 1 mM levamisol is utilised to block non-specific antibody binding sites.

Biochemical ER assessment is not practicable on a substantial number of surgically excised biopsies of DCIS, due to the limited amount of tumour tissue available, and problems in tumour tissue sampling. Tumour sampling of DCIS for assessment of ERICA on frozen tissue is also difficult or often impossible, particularly in situations where assessment of excision margins is important, as in breast conservation treatment, or diagnostic marker biopsies of lesions detected by breast screening. Screening detected cases of DCIS tend to be much smaller than symptomatic cases and biochemical assays are therefore not practicable in the majority of these cases. Direct visualisation of ER by immunocytochemistry allows microscopic assessment of ER in tumour tissue only, and avoids the problems of tumour sampling inherent in biochemical ER receptor assays.

As ER expression is also present in benign mammary epithelial tissue, the presence of significant ER receptor expression in benign breast tissue adjacent to invasive tumours which in some cases are ER negative (Walker *et al.*, 1992) could be a potential cause some false positive biochemical ER assay results.

Our finding of a relatively lower frequency of ER expression in comedo and large cell DCIS as compared to small cell DCIS is consistent with three earlier studies (Barnes & Masood, 1990; Bur *et al.*, 1992; Giri *et al.*, 1989). Barnes and Masood (1990) using DNase I protease pre treatment of archival tissue sections, prior to incubation with ER antibody, found a lower frequency of ER expression in comedo DCIS, as compared to non-comedo DCIS, with all cases of atypical hyperplasia examined showing positive ER staining, albeit with a higher total frequency of ER expression in DCIS in their series. The largest series of ER in DCIS published to date (Bur *et al.*, 1992), used pronase predigestion of archival tissue sections, and also showed a greater frequency of immunohistochemical ER expression in non-comedo DCIS, seen in 55 of 60 tumours (91% of cases), as compared to 20 of 35 cases of comedo DCIS (57% of cases). Bur *et al.* also found a similar relationship of greater frequency of ER expression and small or intermediate cell size, and lack of necrosis. Giri *et al.* (1989) identified ER expression in three of 18 (17% of cases) of comedo DCIS, and 15 of 27 (55% of cases) of cribriform/papillary DCIS giving an overall figure of 42–45% of cases showing 'significant' ER positivity. A further immunohistochemical study of 14 cases of DCIS identified ER expression in eight of 14 tumours (57% of cases) (Malafa *et al.*, 1990). No indication was made in the latter ERICA study as to the type, if any, of enzymatic pre-digestion step employed, and no comment was made regarding ERICA status and histological subtype of DCIS. Another study conducted using frozen tissue also examined ER status by ERICA within the intraductal component of 77 invasive breast carcinomas. A high percentage of tumours showed positive ER expression within the intraductal component, totalling 58 (72%) of cases examined (Ronay *et al.*, 1990).

The largest series of ER in DCIS published to date (Bur *et al.*, 1992) identified an overall ER positivity rate in DCIS and lobular carcinoma *in situ* of 80%, comprising 38 cases of pure DCIS, and 62 cases of invasive carcinoma, with an associated *in situ* component. Bur *et al.* used an equivalent immunohistochemical technique to ourselves, with pronase pre-digestion of tissue sections prior to application of Abbott H222 ER antibody. In this series 25 of 38 (65%) of cases of pure DCIS were ER positive, as compared with 57 of 62 (91%) of cases of invasive carcinoma in which ER immunoreactivity was assessed in the *in situ* component. ER positivity of the *in situ* component was identified in significantly greater number of invasive tumours as compared to pure DCIS.

As 77 of 100 cases in Bur *et al.*'s series were from outside the institution, to allow for potential differences in tissue fixation, all cases of ER negative DCIS or invasive carcinoma with *in situ* disease where ER staining was not present in adjacent normal breast lobules were excluded from the series. It is possible that some cases of ER negative DCIS were excluded from this latter series, most probably large cell and comedo DCIS, particularly as other studies of ER expression in benign tissues adjacent to breast tumours show ER expression in up to 46% of cases in benign tissues adjacent to tumours which are ER negative (Walker *et al.*, 1992). We identified positive ER staining of benign breast acini adjacent to tumour in 36 of the 151 (24%) of cases examined, and 102 of our cases which were ER negative showed no evidence of ER staining in surrounding normal breast ducts. The differences in frequency of ER expression between the various published series may, therefore, be explained by differences in immunohistochemical technique, and differences in cases selected for inclusion.

As all cases in this series were pure DCIS, the differences in frequency of ER expression may be, at least in part, due to the fact that all our cases were pure ductal carcinoma *in situ*, with lobular carcinoma *in situ* and intraduct components of invasive carcinomas being excluded. This may well reflect differences in the biology of pure *in situ* DCIS, as compared to tumours that have undergone clonal selection to form a more aggressive tumour, prior to development of invasive disease (Nowell, 1976).

No significant associations of ER expression and DNA aneuploidy could be identified in our study. There was a significant association of higher S-phase fraction and positive ER status, this is difficult to explain, given our current knowledge of the biology of breast cancer. No other study has examined the relationship of ER and DNA flow cytometry in DCIS to our knowledge. We noted a similar inverse relationship of positive ER status and absence of c-*erb*B-2 overexpression in DCIS, as has also been described by ourselves and others in invasive breast carcinoma (Lovekin *et al.*, 1990).

The major clinical importance of this study is the confirmation in a large series of pure DCIS of earlier more limited studies showing that expression of ER is present in a proportion only, of cases of *in situ* mammary carcinoma, with implications for endocrine therapy of DCIS. This study shows a lower frequency of ER expression than other published series. The potential importance of ER expression in therapy of DCIS will require further prospective and retrospective analyses of ER in DCIS to establish the role of ER status in DCIS in predicting clinical response of DCIS to adjuvant endocrine therapy. Endocrine adjuvant therapy of DCIS is now an integral component of several clinical trials of DCIS, both in the UK (Fentiman, 1992) and the USA.

It is likely, however, that the mechanisms controlling oestrogen receptor mediated signal transduction are complex, and that the absence of immunohistochemically detectable ER may not necessarily preclude patient response to endocrine therapy in some instances, as is the case in invasive breast carcinoma.

Comedo DCIS and subtypes of DCIS showing significant tumour cell necrosis have been shown to have a shorter time to local recurrence after breast conservation treatment. Comedo DCIS and large cell DCIS have a relatively lower frequency of ER expression than small cell DCIS. Comedo DCIS has a higher cellular proliferation fraction as measured by thymidine labelling index (TLI) (Meyer, 1986), or flow cytometric S-phase fraction (Locker *et al.*, 1990). Comedo DCIS shows c-*erb*B-2 oncoprotein overexpression in a higher proportion of cases than non-comedo DCIS (Van de Vijver *et al.*, 1988; Locker *et al.*, 1990).

The confirmation of a relatively higher frequency of ER expression in small cell DCIS and the same inverse relationship of lack of ER expression and c-*erb*B-2 protein overexpression in DCIS as seen in invasive breast carcinoma suggests evidence for homology of ER and c-*erb*B-2 protein expression in both forms of mammary carcinoma. This latter relationship appears conserved in neoplastic progression from *in situ* to invasive disease.

This study addresses one major aspect of ER in DCIS; namely the immunohistochemical expression of oestrogen receptor. Subsequent prospective studies of ER status in DCIS should also take account of the menopausal status of patients, and the role of timing of surgery within the menstrual cycle, both of which may possibly influence the ER status of invasive breast tumours and their response to treatment. This data was not available in our retrospective series. The lower frequency of ER expression in pure DCIS as compared to that in invasive disease may provide an avenue for investigation of the function of oestrogen receptor in the neoplastic progression of mammary carcinoma from *in situ* to invasive malignancy.

### References

AASMUNDSTAD, T.A., HAUGEN, O.A., JOHANNESEN, E., HØE, A.L. & KVINNSLAND, S. (1992). Oestrogen receptor analysis: correlation between enzyme immunoassay and immunohistochemical methods. *J. Clin. Pathol.*, **45**, 125-129.

BAISCH, H., GOHDE, W. & LINDEN, W.A. (1975). Analysis of PCP data to determine the fraction of cells in the various phases of cell cycle. *Radiat. Environ. Biophys.*, **12**, 31-39.

BARNES, R. & MASOOD, S. (1990). Potential value of hormone receptor assay in carcinoma in situ of breast. *Am. J. Clin. Pathol.*, **94**, 533-537.

BUR, M.E., ZIMAROWSKI, M.J., SCHNITT, S.J., BAKER, S. & LEW, R. (1992). Estrogen receptor immunohistochemistry in carcinoma *in situ* of the breast. *Cancer*, **69**, 1174-1181.

CHARPIN, C., MARTIN, P.M., JACQUEMIER, J., LAVAUT, M.N., POURREAU-SCHNEIDER, N. & TOGA, M. (1986). Estrogen receptor immunocytochemical assay (ERICA): computerized image analysis system, immunoelectron microscopy, and comparisons with estradiol binding assays in 115 breast carcinomas. *Cancer Res.*, **46** (Suppl), 4271-4277.

CHENG, L., BINDER, S.W., FU, Y.S. & LEWIN, K.J. (1988). Demonstration of estrogen receptors by monoclonal antibody in formalin-fixed breast tumors. *Lab. Invest.*, **58**, 346-353.

ELIAS, J.M., HEIMANN, A., CAIN, T., MARGIOTTA, M., GALLERY, F. & GOMES, C. (1990). Estrogen receptor localization in paraffin sections by enzyme digestion, repeated applications of primary antibody, and imidazole. *J. Histotechnol.*, **13**, 29-33.

FENTIMAN, I.S. (1990). Treatment of screen detected ductal carcinoma *in situ*: a silver lining within a grey cloud? *Br. J. Cancer*, **61**, 795-796.

FENTIMAN, I.S. (1992). Ductal carcinoma *in situ*: trials needed to decide right treatment. *BMJ*, **304**, 1261-1262.

GIRI, D.D., DUNDAS, S.A.C., NOTTINGHAM, J.F. & UNDERWOOD, J.C.E. (1989). Oestrogen receptors in benign epithelial lesions and intraduct carcinomas of the breast: an immunohistological study. *Histopathology*, **15**, 575-584.

GRAHAM, D.M., JIN, L. & LLOYD, R.V. (1991). Detection of estrogen receptor in paraffin-embedded sections of breast carcinoma by immunohistochemistry and *in situ* hybridization. *Am. J. Surg. Path.*, **15**, 475-485.

HEDLEY, D.W., FRIEDLANDER, M.L. & TAYLOR, I.W. (1985). Application of DNA flow cytometry to paraffin-embedded archival material for the study of aneuploidy and its clinical significance. *Cytometry*, **6**, 327-333.

LOCKER, A.P., HORROCKS, C., GILMOUR, A.S., ELLIS, I.O., DOWLE, C.S., ELSTON, C.W. & BLAMEY, R.W. (1990). Flow cytometric and histological analysis of ductal carcinoma *in situ* of the breast. *Br. J. Surg.*, **77**, 564-567.

LOVEKIN, C., ELLIS, I.O., LOCKER, A., ROBERTSON, J.F.R., BELL, J., NICHOLSON, R., GULLICK, W.J., ELSTON, C.W. & BLAMEY, R.W. (1991). c-erbB-2 oncoprotein expression in primary and advanced breast cancer. *Br. J. Cancer*, **63**, 439-443.

MALAFA, M., CHAUDHURI, B., THOMFORD, N.R. & CHAUDHURI, P.K. (1990). Estrogen receptors in ductal carcinoma *in situ* of breast. *Am. Surg.*, **56**, 436-439.

MCCARTY, K.S., MILLER, L.S., COX, E.B., KONRATH, J. & MCCARTY, K.S. (1985). Estrogen receptor analyses: correlation of biochemical and immunohistochemical methods using monoclonal antireceptor antibodies. *Arch. Pathol. Lab. Med.*, **109**, 716-721.

MEYER, J.S. (1986). Cell kinetics of histologic variants of *in situ* breast carcinoma. *Breast Cancer Res. Treat.*, **7**, 171-180.

NICHOLSON, R.I., BOUZUBAR, N., WALKER, K.J., MCCLELLAND, R., DIXON, A.R., ROBERTSON, J.F.R., ELLIS, I.O. & BLAMEY, R.W. (1991). Hormone sensitivity in breast cancer: influence of heterogeneity of oestrogen receptor expression and cell proliferation. *Eur. J. Cancer*, **27**, 908-913.

NOWELL, P.C. (1976). The clonal evolution of tumor cell populations. *Science*, **194**, 23-28.

PATERSON, D.A., REID, C.P., ANDERSON, T.J. & HAWKINS, R.A. (1990). Assessment of oestrogen receptor content of breast carcinoma by immunohistochemical techniques on fixed and frozen tissue and by biochemical ligand binding assay. *J. Clin. Pathol.*, **43**, 46-51.

PERTSCHUK, L.P., EISENBERG, K.B., CARTER, A.C. & FELDMAN, J.G. (1985). Immunohistologic localization of estrogen receptors in breast cancer with monoclonal antibodies: correlation with biochemistry and clinical endocrine response. *Cancer*, **55**, 1513-1518.

RAYMOND, W.A. & LEONG, A.S.Y. (1990). Oestrogen receptor staining of paraffin embedded breast carcinomas following short fixation in formalin: a comparison with cytosolic and frozen section receptor analyses. *J. Pathol.*, **160**, 295-303.

REMMELE, W., HILDEBRAND, U., HIENZ, H.A., KLEIN, P.J., VIERBUCHEN, M., BEHNKEN, L.J., HEICKE, B. & SCHEIDT, E. (1986). Comparative histological, histochemical, immunohistochemical, and biochemical studies on oestrogen receptors, lectin receptors, and Barr bodies in human breast cancer. *Virchows Arch. (A)*, **409**, 127-147.

ROBERTSON, J.F.R., BATES, K., PEARSON, D., BLAMEY, R.W. & NICHOLSON, R.I. (1992). Comparison of two oestrogen receptor assays in the prediction of the clinical course of patients with advanced breast cancer. *Br. J. Cancer*, **65**, 727-730.

RONAY, G., SPONSEL, N. & TULUSAN, A.H. (1990). Duktales carcinoma in situ (DCIS): steroidrezeptoren und proliferationskinetik. *Pathologe*, **11**, 327-331.

SHINTAKU, I.P. & SAID, J.W. (1987). Detection of estrogen receptors with monoclonal antibodies in routinely processed formalin-fixed paraffin sections of breast carcinoma. Use of DNase pretreatment to enhance sensitivity of the reaction. *Am. J. Clin. Pathol.*, **87**, 161-167.

SNEAD, D.R.J., BELL, J.A., DIXON, A.R., NICHOLSON, R.I., ELSTON, C.W., BLAMEY, R.W. & ELLIS, I.O. Methodology of immunohistological detection of oestrogen receptor in human breast carcinoma in formalin fixed paraffin embedded tissue: A comparison with frozen section methodology. *Histopathology* (in press).

TODD, J.H., DOWLE, C., WILLIAMS, M.R., ELSTON, C.W., ELLIS, I.O., HINTON, C.P., BLAMEY, R.W. & HAYBITTLE, J.L. (1987). Confirmation of a prognostic index in primary breast cancer. *Br. J. Cancer*, **56**, 489-492.

TZUKERMAN, M., ZHANG, X., HERMANN, T., WILLS, K.N., GRAUPNER, G. & PFAHL, M. (1990). The human estrogen receptor has transcriptional activator and repressor functions in the absence of ligand. *New Biol.*, **2**, 613-620.

VAN DE VIJVER, M.J., PETERSE, J.L., MOOI, W.J., WISMAN, P., LOMANS, J., DALESIO, O. & NUSSE, R. (1988). Neu protein overexpression in breast cancer: association with comedo-type ductal carcinoma in situ and limited prognostic value in stage II breast cancer. *N. Engl. J. Med.*, **319**, 1239-1245.

VENTER, D.J., TUZI, N.L., KUMAR, S. & GULLICK, W.J. (1987). Overexpression of the c-erbB-2 oncoprotein in human breast carcinomas: immunohistological assessment correlates with gene amplification. *Lancet*, **ii**, 69–72.

WALKER, K.J., BOUZUBAR, N., ROBERTSON, J., ELLIS, I.O., ELSTON, C.W., BLAMEY, R.W., WILSON, D.W., GRIFFITHS, K. & NICHOLSON, R.I. (1988). Immunocytochemical localization of estrogen receptor in human breast tissue. *Cancer Res.*, **48**, 6517–6522.

WALKER, R.A., COWL, J., DHADLY, P.P. & JONES, J.L. (1992). Oestrogen receptor, epidermal growth factor receptor and oncoprotein expression in non-involved tissue of cancerous breasts. *The Breast*, **2**, 87–91.

WILBUR, D., WILLIS, J., MOONEY, R.A., FALLON, M.A., MOYNES, R. & DI SANT'AGNESE, P.A. (1992). Estrogen and progesterone receptor detection in archival formalin-fixed, paraffin-embedded tissue from breast carcinoma: a comparison of immunohistochemistry with the dextran-coated charcoal assay. *Mod. Pathol.*, **5**, 79–84.